# Supplementary material for: Interleukin-34 Enhances the Tumor Promoting Function of Colorectal Cancer-Associated Fibroblasts
Source: Cancers (Basel). 2020 Nov 27;12(12):3537. doi: 10.3390/cancers12123537 (PMC7761053; doi:10.3390/cancers12123537)
Supplement: Supplementary file 1 [file cancers-12-03537-s001.pdf]

# Supplementary Material: Interleukin-34 Enhances the Tumor Promoting Function of Colorectal Cancer-Associated Fibroblasts

**Eleonora Franzè, Antonio Di Grazia, Giuseppe Sigismondo Sica, Livia Biancone, Federica Laudisi and Giovanni Monteleone**

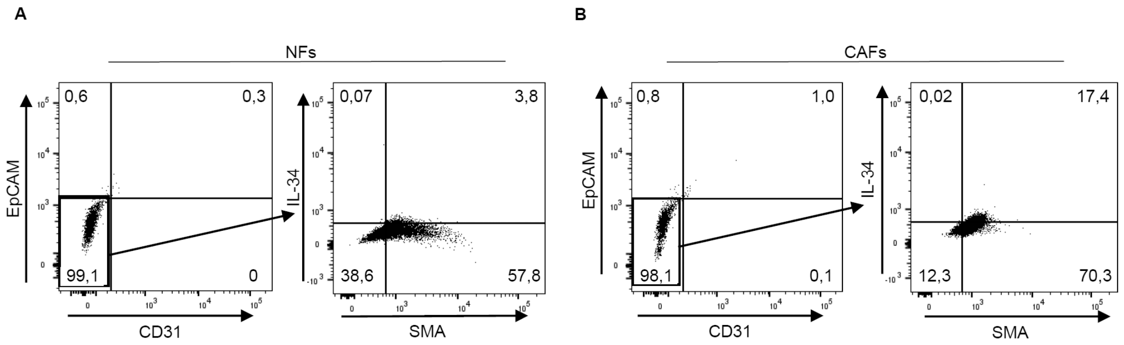


**Figure S1.** CAFs express interleukin-34 (IL-34). (**A**),(**B**). Left panels: representative dot-plots showing expression of CD31 and EpCAM in normal fibroblasts (NFs) (**A**) and cancer-associated fibroblasts (CAFs) (**B**) taken from tumoral and non-tumoral areas of one patient with colon cancer; right panels show expression of $\alpha$-SMA and IL-34 in cells negative for CD31 and EpCAM. One of two experiments in which similar results were obtained is shown.

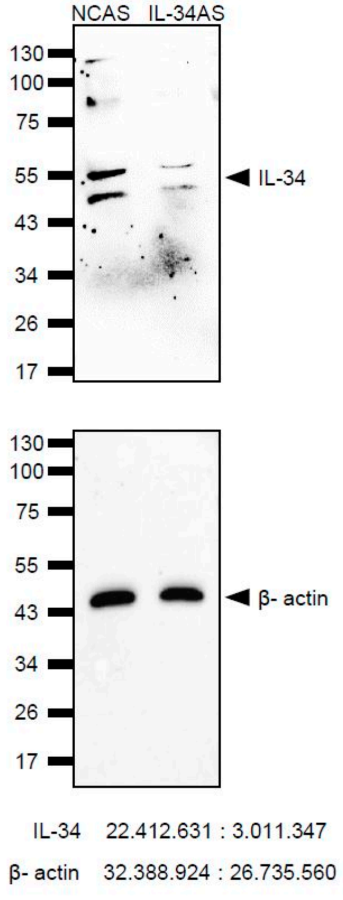


**Figure S2.** Uncropped Western Blot of Figure 3D.

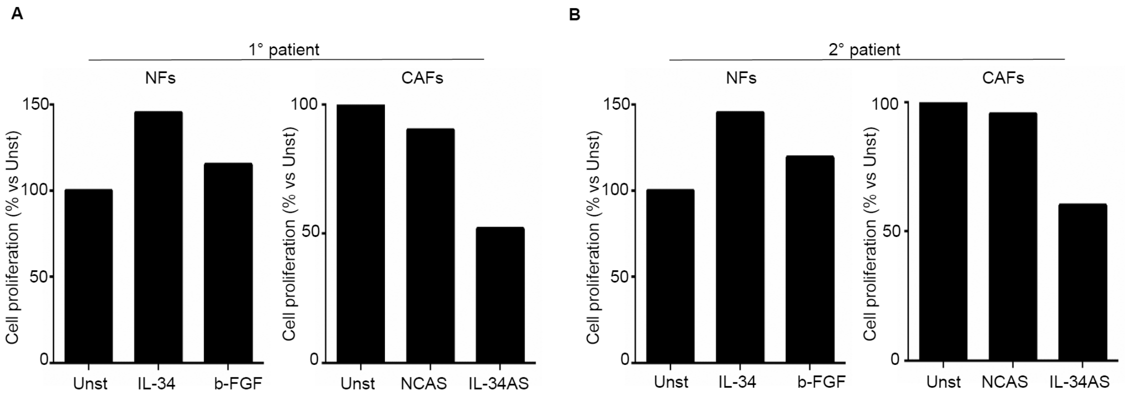


**Figure S3.** IL-34 enhances fibroblast proliferation. (**A**) 1°patient, (**B**), 2° patient. Left panels: serum-starved normal fibroblasts (NFs) were stimulated with IL-34 (50 ng/mL) or basic-fibroblast growth factor (b-FGF) (20 ng/mL) for 48 h. Right panels: cancer-associated fibroblasts (CAFs) were transfected with negative control antisense oligonucleotide (NCAS) or IL-34 antisense oligonucleotide (IL-34 AS) for 48 h. Cell proliferation was evaluated by 5-bromodeoxyuridine (BrdU) proliferation assay kit. Results of two experiments that analyzed cells of two patients are shown. Unst = unstimulated.

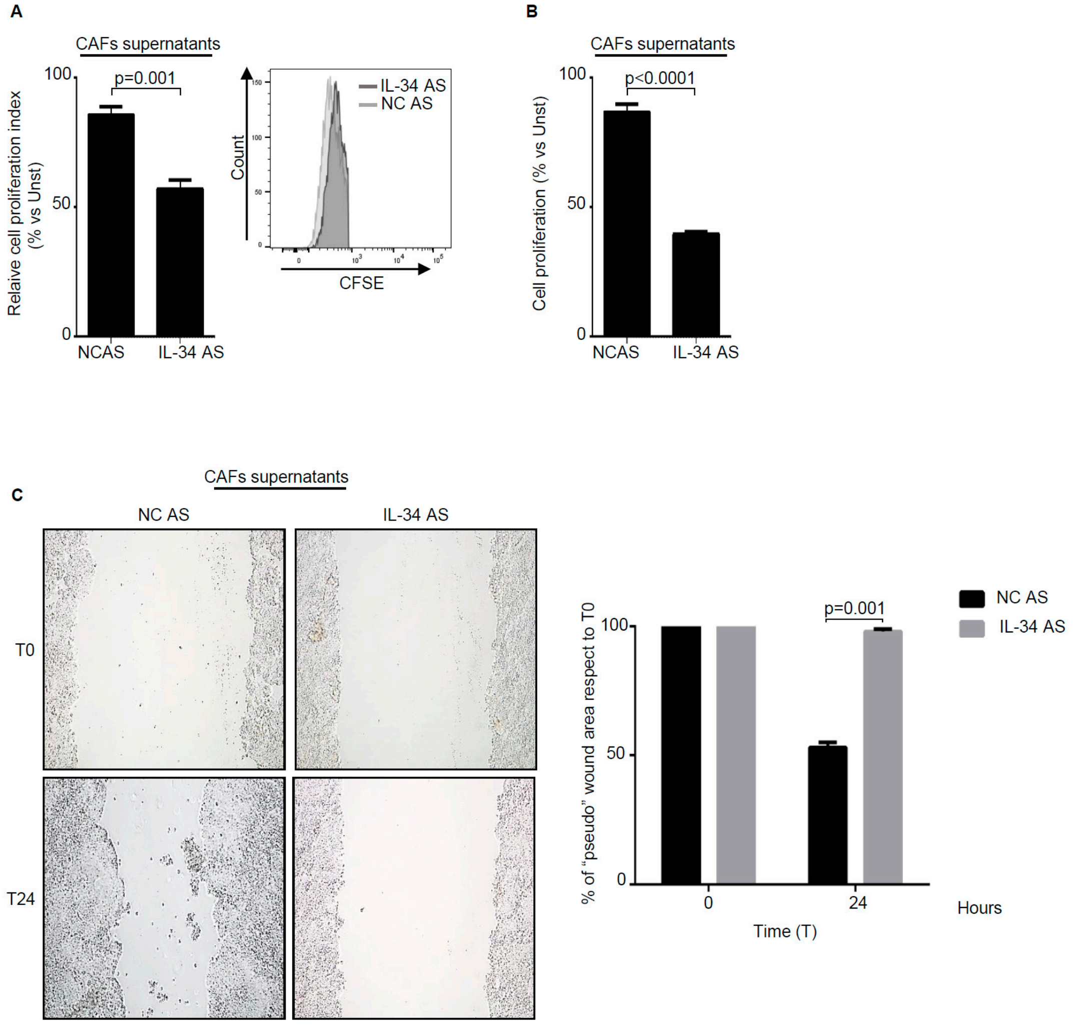


**Figure S4.** IL-34 produced by fibroblasts decreases HT-29 cell proliferation and migration. (**A**),(**B**) HT-29 cells were incubated with supernatants of CAFs previously transfected with either negative control

antisense oligonucleotide (NCAS) or IL-34 antisense oligonucleotide (IL-34 AS) for 24 h. HT-29 cell proliferation was evaluated after 48 h by flow cytometry, and proliferation index was calculated with Modfit LT (Verity Software House, Inc., Topsham, ME, USA) (**A**) or by a 5-bromodeoxyuridine (BrdU) proliferation assay kit (Roche Diagnostics, Monza, Italy) (**B**). Data indicate mean ± SEM of four independent experiments in which cells of four patients were analyzed. Right insets in (**A**): representative histograms showing the expression of CFSE in HT-29 analyzed by flow cytometry. (**C**). Representative images of "pseudo" wound in monolayer of HT-29 cells treated with supernatants of CAFs previously transfected with either negative control antisense oligonucleotide (NCAS) or IL-34 antisense oligonucleotide (IL-34 AS) for 24 h. Cells were photographed at the time of scratch (T0) and examined for cell migration after 24 h from the specific stimulation. Right panel shows the % of "pseudo" wound area in a monolayer of HT-29 cells at the specific time point (T) with respect to area in T0 (defined as 100%), and the data are expressed as mean ± SEM of three independent experiments in which cells of three patients were analyzed.

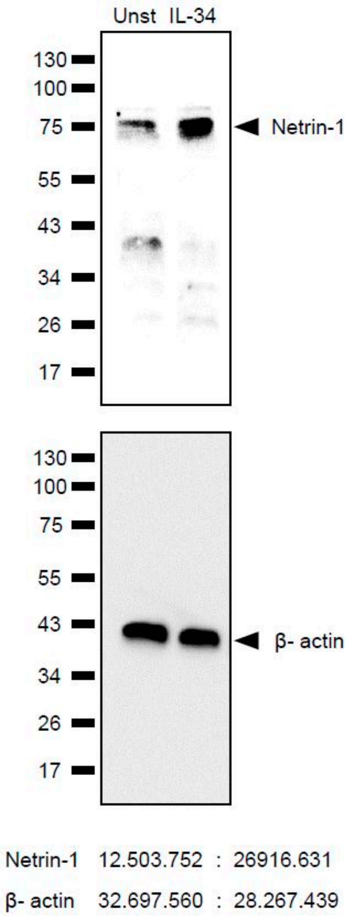


**Figure S5.** Uncropped Western Blot of Figure 7A.

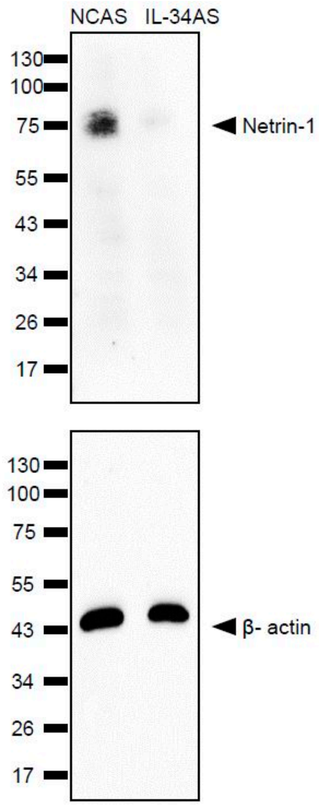


Netrin-1 43492476 : 4469396

β- actin 32.112.146 : 26.410.004

**Figure S6** Uncropped Western Blot of Figure 7C.

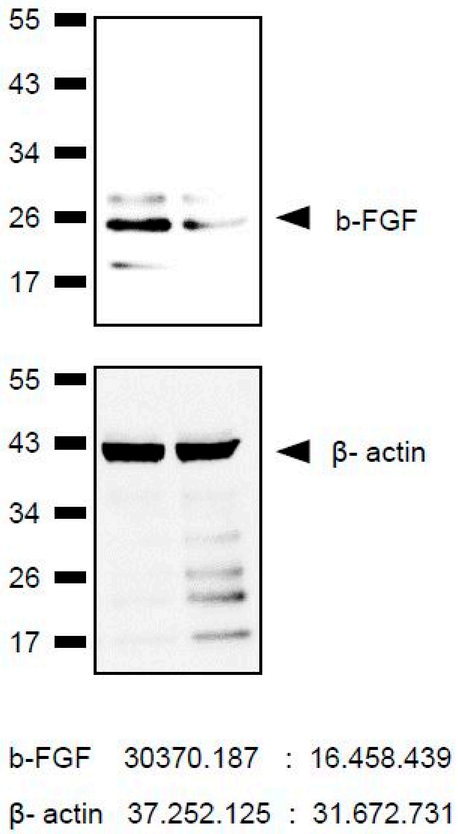


**Figure S7.** Uncropped Western Blot of Figure 7D.

**Publisher's Note:** MDPI stays neutral with regard to jurisdictional claims in published maps and institutional affiliations.

© 2020 by the authors. Submitted for possible open access publication under the terms and conditions of the Creative Commons Attribution (CC BY) license (http://creativecommons.org/licenses/by/4.0/).
